# Redox status of patients before cardiac surgery

**DOI:** 10.1080/13510002.2017.1418620

**Published:** 2017-12-19

**Authors:** Anna Katharina Schuh, Babak Sheybani, Esther Jortzik, Bernd Niemann, Jochen Wilhelm, Andreas Boening, Katja Becker

**Affiliations:** a Biochemistry and Molecular Biology, Interdisciplinary Research Center, Justus Liebig University, Giessen, Germany; b Clinic for Heart, Pediatric Heart and Vascular Surgery, Faculty of Medicine, UKGM, Giessen, Germany; c Excellence Cluster Cardio-Pulmonary System, Justus Liebig University, Giessen, Germany

**Keywords:** Cardiac surgery, coronary artery disease, calcium channel blocker, glutathione, pulmonary hypertension, reactive oxygen species

## Abstract

**Objectives:** Redox regulation plays a crucial role in balancing the cardiovascular system. In this prospective study we aimed to identify currently unknown correlations valuable to cardiovascular research and patient management.

**Methods:** Blood samples from 500 patients were collected directly before cardiosurgical interventions (Ethics Committee reference number 85/11). Four central redox parameters were determined together with about 30 clinical, anthropometric, and metabolic parameters.

**Results:** Creatinine levels and pulmonary hypertension were significant predictors of the total antioxidant status (TAOS) in the patients; total glutathione levels were linked to C-peptide, and creatinine, gender, and ventricular arrhythmia influenced nitrate/nitrite levels. Notably, significant interactions were found between medication and redox parameters. Calcium channel blockers (CCBs) were positive predictors of total glutathione levels, whereas angiotensin-converting enzyme inhibitors and CCBs were negative predictors of NOx levels. Age showed the highest correlation with the duration of the intensive care stay, followed by NOx levels, creatinine, TAOS, and C-reactive protein.

**Discussion:** In this prospective study we determined multiple correlations between redox markers and parameters linked to cardiovascular diseases. The data point towards so far unknown interdependencies, particularly between antihypertensive drugs and redox metabolism. A thorough follow-up to these data has the potential to improve patient management.

**Abbreviations:** A: absorption; ΔA: absorption difference; ABTS: 2,2′-azino-di(3-ethylbenzothiazoline sulfonate); ACE: angiotensin-converting enzyme; AO: antioxidant; ARB: angiotensin receptor blocker; BMI: body mass index; CAD: coronary artery disease; CCB: calcium channel blocker; CDC: coronary heart diseases; COPD: chronic obstructive pulmonary disease; CRP: C-reactive protein; CVD: cardiovascular diseases; Cu-OOH: cumene hydroperoxide; D: dilution factor; DAN: 2,3-diaminonaphtalene; DMSO: dimethylsulfoxide; DNA: deoxyribonucleic acid; DTNB: 5,5-dithiobis(2-nitrobenzoate); *ϵ*: extinction coefficient; EDRF: endothelium-derived relaxing factor; fc: final concentration; GPx: glutathione peroxidases; (h)GR: (human) glutathione reductase; GSH: (reduced) glutathione; GSSG: glutathione disulfide; GST: glutathione-S-transferase; Hb: hemoglobin; HDL: high-density lipoprotein; Hk: hematocrit; H_2_O_2_: hydrogen peroxide; ICS: intensive care stay; LDH: lactate dehydrogenase; LDL: low-density lipoprotein; MI: myocardial infarction; NED: *N*-(1-naphthyl)-ethylendiamine-dihydrochloride; NOS: nitric oxide synthase; NOx: nitrate/nitrite; NR: nitrate reductase; PBS: phosphate buffered saline; PCA: principle component analysis; PH: pulmonary hypertension; ROS: reactive oxygen species; RNS: reactive nitrogen species; RT: room temperature (25°C); SA: sulfanilamide; SOD: superoxide dismutase; SSA: sulfosalicylic acid; TAC: total antioxidant capacity; TAOS: total antioxidant status; TEAC: trolox equivalent antioxidative capacity; TG: triglycerides; tGSH: total glutathione; TNB-: 2-nitro-5-thiobenzoate; U: unit; UV: ultraviolet; VA: volume activity; Wc: working concentration; WHR: waist-hip ratio.

## Introduction

A thoroughly tuned balance between oxidative challenge and antioxidant defense is essential for maintaining functionality and integrity of the cardiovascular system, including cardiac myocytes and endothelial cells [1,2]. Major sources of oxidative stress in the cardiovascular system are NAD(P)H oxidase (in membrane complexes), mitochondrial cytochromes, xanthine oxidoreductase, reactive nitrogen species (NOx), and hemoglobin [3]. Furthermore, redox stress has been shown to be a part of the pathophysiology of cardiovascular diseases (CVDs) such as atherosclerosis, ischemia/reperfusion injury, cardiomyopathies, cardiac hypertrophy, hypertension, ventricular remodeling, and congestive heart failure [2,4,5]. As a result, close links between oxidative stress, vascular inflammation, endothelial dysfunction, and cardiovascular risk factors have been revealed [6,7]. To support cellular redox homeostasis, also under enhanced oxidative stress, supplementation with antioxidants such as coenzyme Q10 (CoQ10), beta-carotene, lycopene, quercetin, resveratrol, vitamins C and E, and other bioactive compounds is being intensely discussed [2,8,9]. Altered redox parameters are proposed to serve as risk factors for CVDs [10]; however, they might also be valuable predictors for complications and the outcome of cardiosurgical interventions.

Among the redox parameters that can be determined in the peripheral blood of patients, total antioxidant status (TAOS), total glutathione concentrations, glutathione peroxidase activity, and both nitrite and nitrate concentrations are of particular interest in the context of CVDs. TAOS, often called total antioxidant capacity, is equivalent to the capacity of a sample (plasma, food, tissue homogenate, or chemical compounds) to intercept or neutralize a synthetic redox-active molecule, the ABTS radical, in comparison to a reference compound [11]. Several components of blood plasma such as total protein, uric acid, bilirubin, carotinoids, tocopherols, and ascorbic acid contribute to the antioxidant capacity summarized in the TAO status [12,13]. Glutathione, as a major thiol antioxidant and redox buffer in cells, is involved in redox regulation, gene expression, signal transduction, and apoptosis [14]. Under oxidative stress, the high GSH:GSSG ratio can be diminished, which may be accompanied by protein glutathionylation, changes in glutathione compartmentalization, and glutathione export from the cell [15]. Therefore, the glutathione blood concentration, especially the ratio of its reduced and oxidized forms, is a valuable parameter for determining the redox status of a patient [3]. Glutathione peroxidases (GPx) are enzymes that contain selenium and prevent the formation of free radicals by reducing peroxides to alcohols [16]. The GPx family comprises eight isoenzymes that use different substrates and glutathione as an electron donor [17]. NO is a radical gas and therefore very unstable. In the presence of molecular oxygen, it spontaneously autoxidizes to nitrite (${\text{NO}}_{2}^{-}$) and nitrate (${\text{NO}}_{3}^{-}$), with nitrate as the major oxidative metabolite [18]. Today many physiological functions of NO with major importance in the context of CVDs are known: it mediates vasodilatation, thus influencing blood pressure and blood flow, inhibits platelet aggregation, and promotes neurotransmission and host defense against infectious agents. Due to its short lifespan, NO has autocrine and paracrine functions. However, its more stable products ${\text{NO}}_{2}^{-}$ and ${\text{NO}}_{3}^{-}$ are considered mediators of endocrine functions [19]. During physiological hypoxia, the inorganic anions ${\text{NO}}_{2}^{-}$ and ${\text{NO}}_{3}^{-}$ are reduced in blood and tissues in order to form bioactive NO and therefore supposedly function as storage pools for it [20]. Regression of oxidative stress by targeting endothelial nitric oxide synthase is being discussed as a drug target for CVDs [21].

For this prospective study, we asked whether there are relationships between the redox status of patients directly before cardiosurgical interventions and other clinically and metabolically relevant parameters including gender, age, anthropometric parameters, lipid metabolites, drug intake such as angiotensin-converting enzyme (ACE) inhibitors, AT-II receptor blockers, beta-blockers, and calcium channel blockers (CCBs), CVDs such as coronary heart disease, chronic obstructive pulmonary disease (COPD), and pulmonary hypertension (PH), and the duration of the treatment at the intensive care unit. If such relationships exist, they might be employed to improve patient management and preventive treatment.

## Materials and methods

### Patients

Patients were recruited from September 2011 to April 2013 at the Department of Cardiovascular Surgery, Justus Liebig University, in Giessen, Germany. Inclusion criteria were defined as follows: indication for cardiac surgery, patients over 30 years of age, adequate German language skills, and capacity to provide fully informed written consent. Exclusion criteria were the following: emergencies, if an explanation of the potential risks of the study were not possible, patients whose body mass could not be measured, and drug and alcohol abuse. The ethics committee of the Medical Faculty, Justus Liebig University, Giessen, approved the ethical aspects of the study (reference number 85/11).

### Materials

Venous EDTA blood samples from 500 patients were taken in the morning directly before surgery and treated as previously described [22,23]. Briefly, for glutathione determination, 200 µl of the blood sample was immediately mixed with 600 µl of 5% sulfosalicylic acid (Merck, Darmstadt, cat. no. 1.00691.0100). After vortexing (2 times for 10 s), the sample was centrifuged at 10,000 g for 10 min at 4°C. The supernatant was stored in order to determine total glutathione levels. For determining glutathione peroxidase activity, aliquots of whole blood were stored in vials. The remaining blood was carefully centrifuged (10 min, 2,500 g, 4°C), and the plasma was pipetted into fresh vials in order to determine TAOS and NOx. All samples were immediately frozen at −80°C. Unless otherwise stated, all reagents were obtained from Roth (Karlsruhe), Sigma (Steinheim), Serva (Heidelberg), Biomol (Hamburg), and Merck (Darmstadt) and were of the highest purity available. Recombinant human glutathione reductase was produced as described previously [24].

### Determination of TAOS

TAOS was determined with the ABTS assay according to Miller et al. [25], which is based on the ability of antioxidants to prevent ABTS oxidation, and was adapted to the 96-well plate format. Metmyoglobin is oxidized to ferryl myoglobin with hydrogen peroxide (H_2_O_2_). There, ferryl myoglobin oxidizes ABTS to the blue–green ABTS+ radical, which can be measured at 600 nm. In the presence of antioxidants, radical formation is suppressed, allowing TAOS to be calculated in comparison to a standard (Trolox).

First, 106 μl of Mix 1, containing 6 μM metmyoglobin (Serva, Heidelberg, cat. no. 29895), 600 μM ABTS (Sigma, Steinheim, cat. no. A1888), and 0.05% anti-foam (Sigma, Steinheim, cat. no. A8582) in PBS buffer was pipetted into a 96-well plate (Greiner, half-area), and 4 μl of plasma, 0.5 mM Trolox standard (Sigma, Steinheim, cat. no. 238813), or water was added, and background absorbance was measured at 600 nm. 10 μl of a freshly prepared H_2_O_2_ solution was added to a final concentration of 242 μM and mixed immediately. After exactly 3 min, absorbance was measured again. In order to obtain reliable data, it is very important to measure all samples after the exact same incubation time and at the same temperature (25°C). TAOS was calculated with the following equations:

$$\text{TAOS}=f*((\Delta A_{\mathrm{blank}}-A_{\mathrm{sample}})\left[\mathrm{mM}\right]$$

$$f*\text{concentration of the standard}/(\Delta A_{\mathrm{blank}}-\Delta A_{\mathrm{standard}}).$$

### Determination of total glutathione concentrations in blood

The determination of total glutathione concentrations was based on the DTNB glutathione recycling assay [26,22] and was optimized for the microplate reader. Reduced glutathione reacts with DTNB, forming the yellow TNB^−^ radical and oxidized glutathione. Oxidized glutathione can be reduced via glutathione reductase (GR) to GSH, which immediately reacts with DTNB again. This principle enables determination of both reduced and oxidized glutathione in a sample. Absorbance of the TNB^−^ chromophore can be measured at 412 nm, and the charge in absorbance per minute is proportional to the concentration of total glutathione.

118 µl of stock buffer (143 mM sodium phosphate, 6.3 mM EDTA, pH 7.5) containing 400 µM NADPH (Biomol, Hamburg, cat. no. 16156.500) and 0.015% anti-foam was pipetted into a 96-well plate (Greiner, half-area). 2 μl of sample, sulfosalicylic acid (blank) or standard, and 10 µl of a DTNB (Roth, Karlsruhe, cat. no. 6334) solution were added to a final concentration of 600 µM. After 10 min of incubation, 10 µl of hGR in stock buffer (final concentration 0.2 U/ml) was added, gently mixed, and immediately measured over 3 min at 412 nm. For calculating total glutathione concentrations, the absorbance per minute of the linear phase of the reaction (typically from 50 to 180 s) was related to a standard curve (0–5 µM GSH; Sigma, Steinheim, cat. no. G6529). The concentration in erythrocytes was calculated on the basis of the hematocrit and whole blood total glutathione.

### Glutathione peroxidase activity

The assay used in our study was based on Beutler’s method for determining glutathione peroxidase activity [27]. Glutathione peroxidases reduce peroxides to alcohols, using GSH as an electron donor. GSSG is recycled back to GSH by GR and NADPH. The activity of GPx is the rate-limiting factor in this reaction and is proportional to the decrease of NADPH absorbance, which can be measured at 340 nm.

First, 35 µl of GPx buffer (100 mM Tris, 0.5 mM EDTA, pH 8.0) containing hGR (1.9 U/ml), NADPH (200 µM) and anti-foam (0.05%) were pipetted into a 96-well plate. Blood samples were diluted with GPx buffer (dilution factor 20), and red blood cells were lysed with digitonin (40 µg/ml, dilution factor 5; Sigma, Steinheim, cat. no. D5628) and added to the plate. After 2 min of incubation, cumene hydroperoxide (Sigma, Steinheim, cat. no. C0524) was added to a final concentration of 1 mM. Absorbance was measured at 340 nm over 3 min at 25°C. Enzyme activity (U/ml) was calculated using an *ϵ* of 6.22 mM^−1^ cm^−1^.

### Nitrate/nitrite determination

The Griess colorimetric assay [28] with slight modifications was used to determine plasma nitrate and nitrite concentrations as the stabile end products of nitric oxide. The most important modification was the introduction of a protein precipitation step using zinc sulfate as also recommended by other studies, including Sastry et al. [29]. Under acidic conditions, nitrite forms nitrous acid, which reacts with sulfanilamide (4-aminobenzolsulfanilamide; Sigma, Steinheim, cat. no. 59251) to form its diazonium salt, which reacts with NED (N-(1-naphthyl)ethylenediamine-dihydrochloride, Santa Cruz Biotechnology, Dallas, cat. no. sc-203148), forming a pink chromophore that absorbs at 540 nm. To convert nitrate to nitrite, 40 µl of a reaction mixture (50 mM HEPES, 5 µM FAD, 100 µM NADPH, 0.2 U/ml nitrate reductase) was mixed with 40 µl of diluted plasma and incubated for 30 min at 37°C. To remove NADPH, lactate dehydrogenase (14 U/ml) and pyruvate (9 mM) were added and incubated for another 10 min. For deproteinization, 40 µl of ZnSO_4_ (300 mg/ml) was added, mixed thoroughly, and centrifuged (8 min, 1,200 g). Afterwards 70 µl of the supernatant was mixed with 23 µl of Griess reagent that contained equal volumes of sulfanilamide and NED in phosphoric acid. After 10 min of incubation, absorbance was measured at 543 nm. Background levels were measured by using phosphoric acid instead of Griess reagent. NOx levels were calculated from the absorbance per minute related to a standard curve with KNO_3_ (0 to 25 µM).

### Other parameters

All other parameters were assessed using standard methods employed in clinical chemistry as established at the Department of Clinical Chemistry, University Hospital Giessen.

### Statistics

Basic descriptive statistics (5-point-summary, mean, standard deviation, and frequencies for categorical factors) for all parameters were calculated with Microsoft Excel. All further statistical analyses were done using the open source software R [30]. An exploratory analysis revealed an approximate log-normal distribution of some parameters (NOx, C-peptide, HDL, triglycerides (TG), C-reactive protein (CRP), creatinine, leucocytes, and intensive care stay (ICS)), which was considered in subsequent analyses by using the log-transformed values that approximate a normal distribution (this was the case for NOx). The distributions of the other variables were also checked. The following variables showed a log-logmal distribution and were therefore analyzed after log transformation: C-peptide, HDL, TG, CRP, creatinine, leucocytes, and ICS. The Pearson correlation coefficients were calculated for all pairs of parameters. The hierarchical cluster of the parameters was analyzed using (1−*r*²) as a distance metric (where *r* is the Pearson’s coefficient of correlation), and agglomeration was done based on complete linkage.

For the principal component analysis (PCA), categorical variables were coded −1 for ‘no’ and + 1 for ‘yes’. All data were centered and normalized. For each of the four redox parameters (TAOS, tGSH, GPx, NOx), an independent multiple linear regression model was designed. All other parameters served as independent variables, including all pairwise interactions. The models were simplified using stepwise selection of predictors based on Akaike’s information criterion.

## Results

### Numerical parameters, categorical parameters, and descriptive statistics

Peripheral blood from 500 patients was taken at one time point in the morning directly before a cardiosurgical intervention. In this blood sample, total glutathione concentrations, glutathione peroxidase activity, plasma TAOS, as well as plasma nitrite and nitrate were determined. Gender, age, anthropometric parameters, important clinical chemistry parameters, and the duration of stay at the intensive care unit after surgery were also monitored. Table 1 shows a 5-point-summary, mean, standard deviation, and reference values for all numerical parameters determined.

**Table 1.** Number of cases, range, mean, standard deviation, and reference values for all numeric parameters determined in the study. Reference values for redox parameters are discussed in the text.

Parameter	*N*	Range	Mean ± SD	1st Q/Median/3rd Q	Reference
TAOS [µM]	469	175–895	555 ± 107	483/550/617	see text
tGSH in whole blood [mM]	500	0.11–1.11	0.61 ± 0.19	0.48/0.62/0.76	see text
tGSH in erythrocytes [mM]	497	0.34–2.83	1.52 ± 0.48	1.18/1.56/1.86	see text
GPx [U/ml whole blood]	500	5.69–32.3	15.6 ± 4.26	12.65/15.45/18.41	see text
GPx [U/mg Hb]	497	0.04–0.22	0.11 ± 0.03	0.09/0.11/0.13	see text
NOx [µM]	484	8.79–183	35.9 ± 25.0	20.9/28.5/41.6	see text
Age [years]	497	22–89	68.6 ± 10.6	63/71/76	–
BMI	494	16.9–43.4	28.3 ± 4.40	25.4/28.0/30.8	18.5–24.9
WHR	495	0.78–1.20	1.00 ± 0.06	0.97/1.02/1.04	< 0.85 (w); <1.0 (m)
C-peptide [ng/ml]	493	0.49–21.1	2.46 ± 1.55	1.61/2.16/2.88	0.9–4.0
HDL [mg/dl]	495	13–104	42.3 ± 12.4	34/40/49	40–80
LDL [mg/dl]	496	10–242	104 ± 36.3	78/100/127	65–150
Triglycerides [mg/dl]	497	43–858	148 ± 95.4	94/127/169	50–245
CRP [mg/l]	496	0.50–296	8.97 ± 19.8	0.78/2.9/8.70	< 1
Creatinine [mg/dl]	497	0.4–6.9	1.04 ± 0.56	0.8/0.9/1.1	0.6–1.3
Leucocytes [giga/l]	496	2.2–19.6	7.6 ± 2.22	6.1/7.3/8.85	3.9–10.2
ICS [days]	496	1–50	3.68 ± 4.90	2/2/4	–

Abbreviations used: BMI: body mass index; CRP: C-reactive protein; GPx: glutathione peroxidases; tGSH: total (reduced and oxidized) glutathione; HDL: high-density lipoprotein; ICS: intensive care stay; LDL: low-density lipoprotein; NOx: reactive nitrogen species; TAOS: total antioxidant status; WHR: waist–hip ratio.

**Figure 1.**
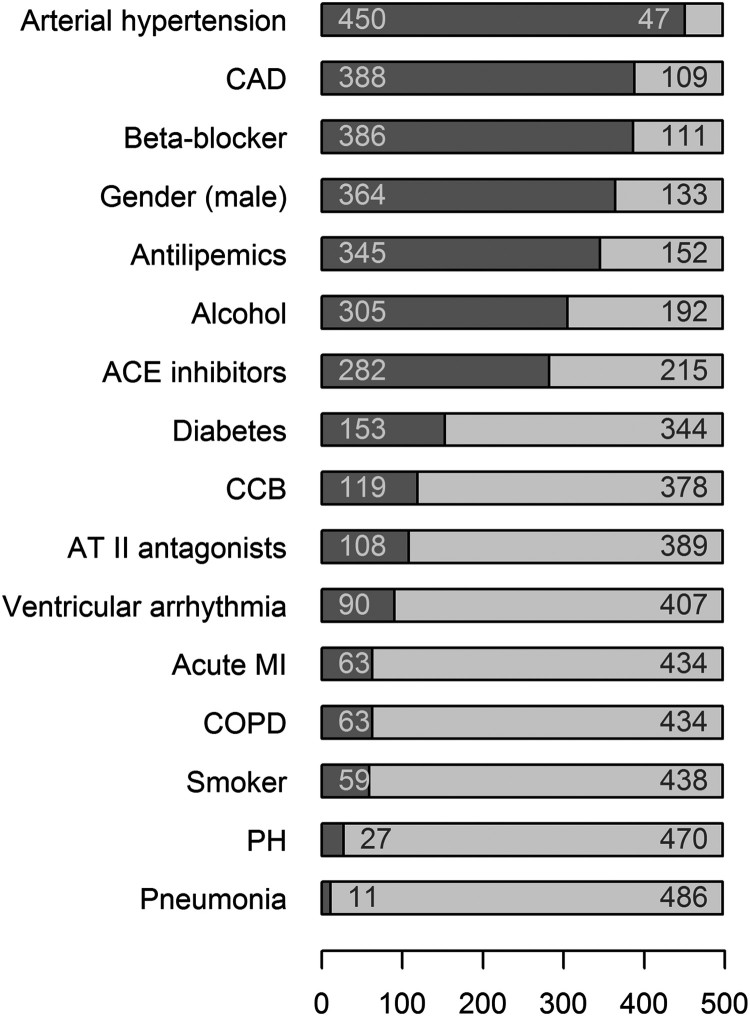
Distribution of the categorical characteristics of the patients. The number of patients was 497. The figure shows for each parameter the number of positive (i.e. arterial hypertension 450) and negative (i.e. 47) cases. Abbreviations used: ACE: angiotensin-converting enzyme; CAD: coronary artery disease; CCB: calcium channel blocker; COPD: chronic obstructive pulmonary disease; MI: myocardial infarction; PH: pulmonary hypertension.

Moreover, central categorical parameters were monitored for the patients. They include gender, alcohol consumption, cigarette smoke, and the intake of CCBs, beta blockers, ACE inhibitors, angiotensin receptor blockers (ARBs, angiotensin II receptor subtype 1 antagonists, sartans), and antilipemics, as well as the diagnosis of arterial hypertension, coronary artery disease (CAD), ventricular arrhythmia, diabetes, acute myocardial infarction (MI), COPD, PH, or pneumonia. Figure 1 shows the distribution of these categorical characteristics of the patients with the number of cases for each parameter.

**Figure 2.**
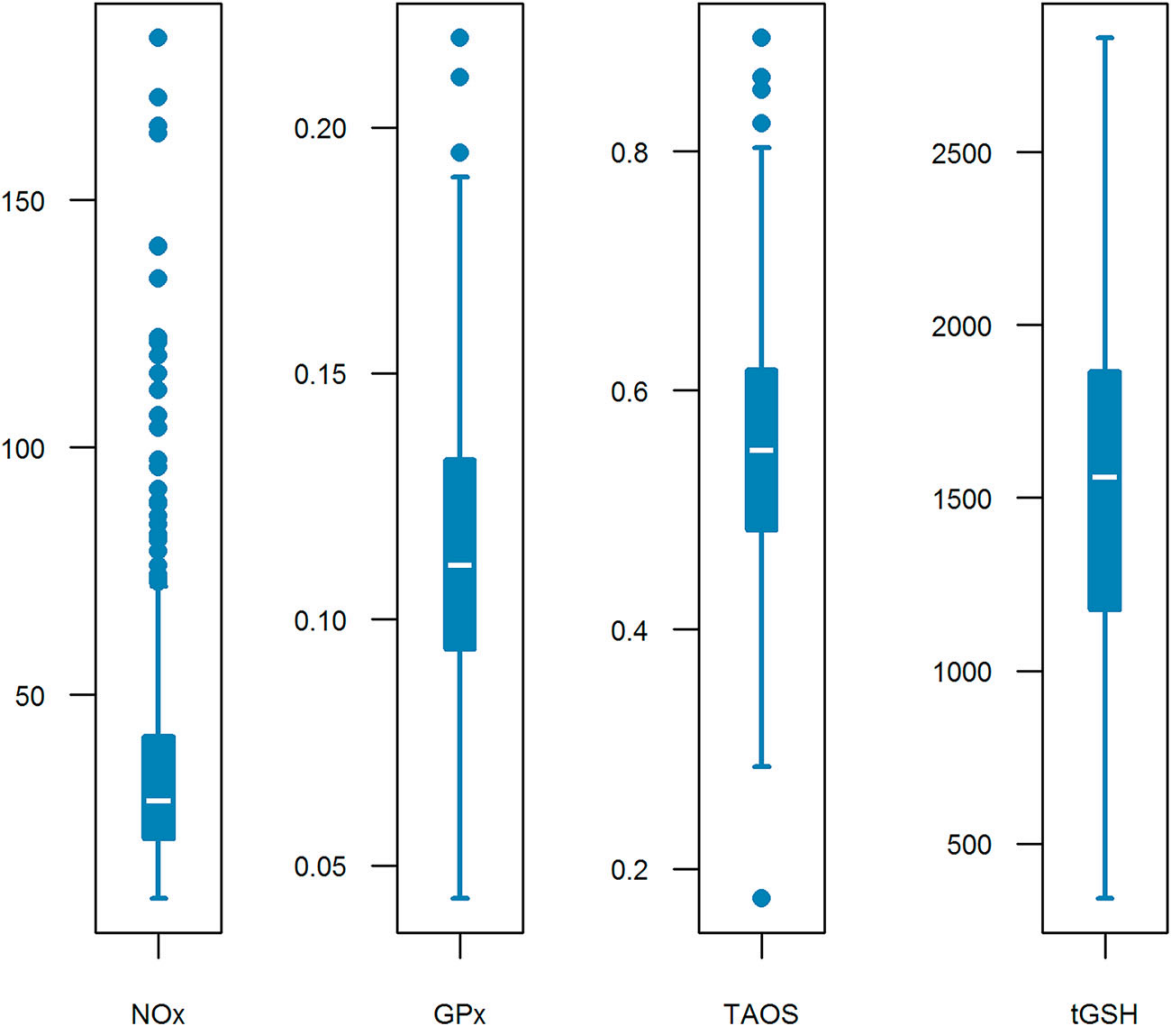
Box-whisker plots of the four redox parameters. Abbreviations used: GPx: glutathione peroxidases [U/ml]; NOx: nitrate/nitrite [µM]; TAOS: total antioxidant status [mM]; tGSH: total (reduced and oxidized) glutathione [µM].

Furthermore, box-and-whisker plots for the four redox parameters determined are provided in Figure 2. The plots indicate data distribution. GPx, TAOS, and tGSH show a rather symmetric distribution, which is approximately normal. The distribution of NOx is clearly right skewed, whereas log(NOx) is normally distributed.

### PCA, correlation, and cluster analysis

**Figure 3.**
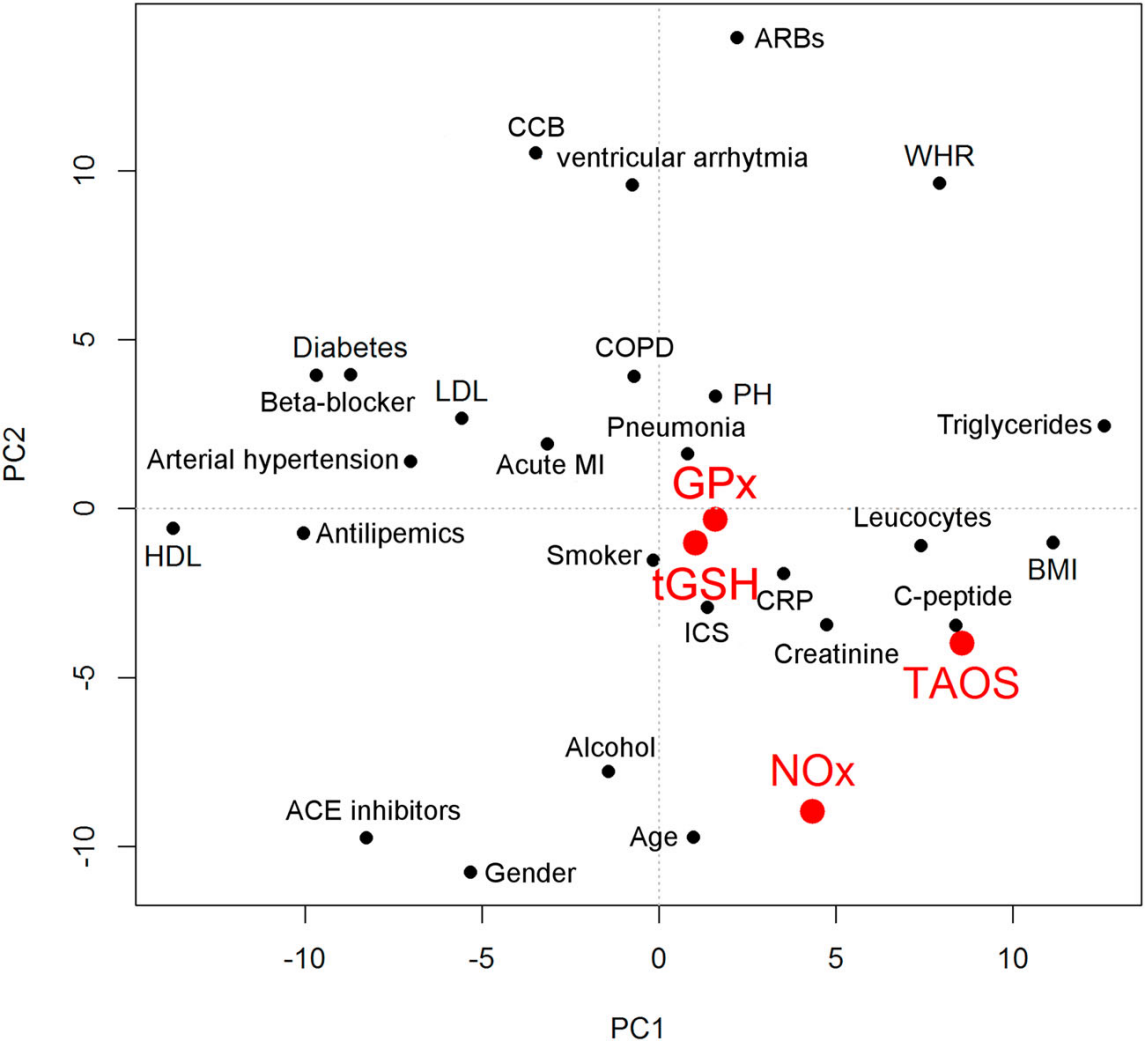
Principal component analysis (PCA) of all parameters. Redox parameters are shown in red. Abbreviations used: ACE: angiotensin-converting enzyme; ARB: angiotensin receptor blocker; BMI: body mass index; CCB: calcium channel blocker; COPD: chronic obstructive pulmonary disease; CRP: C-reactive protein; GPx: glutathione peroxidases; HDL: high-density lipoprotein; ICS: intensive care stay; LDL: low-density lipoprotein; MI: myocardial infarction; NOx: nitrate/nitrite; PH: pulmonary hypertension; TAOS: total antioxidant status; tGSH: total glutathione; WHR: waist–hip ratio.

The major purpose of this study was to evaluate relationships between redox parameters and other clinical and metabolic parameters assessed. The PCA, shown in Figure 3, gives initial insights. The biplot shows waist-hip ratio (WHR), triglycerides (TG), and pulmonary hypertension (PH) in the upper right quadrant. Arterial hypertension, beta-blocker, and acute MI are close together. Likewise CRP and leucocytes are located together, as are PH and COPD as well as smoking and pneumonia.

**Figure 4.**
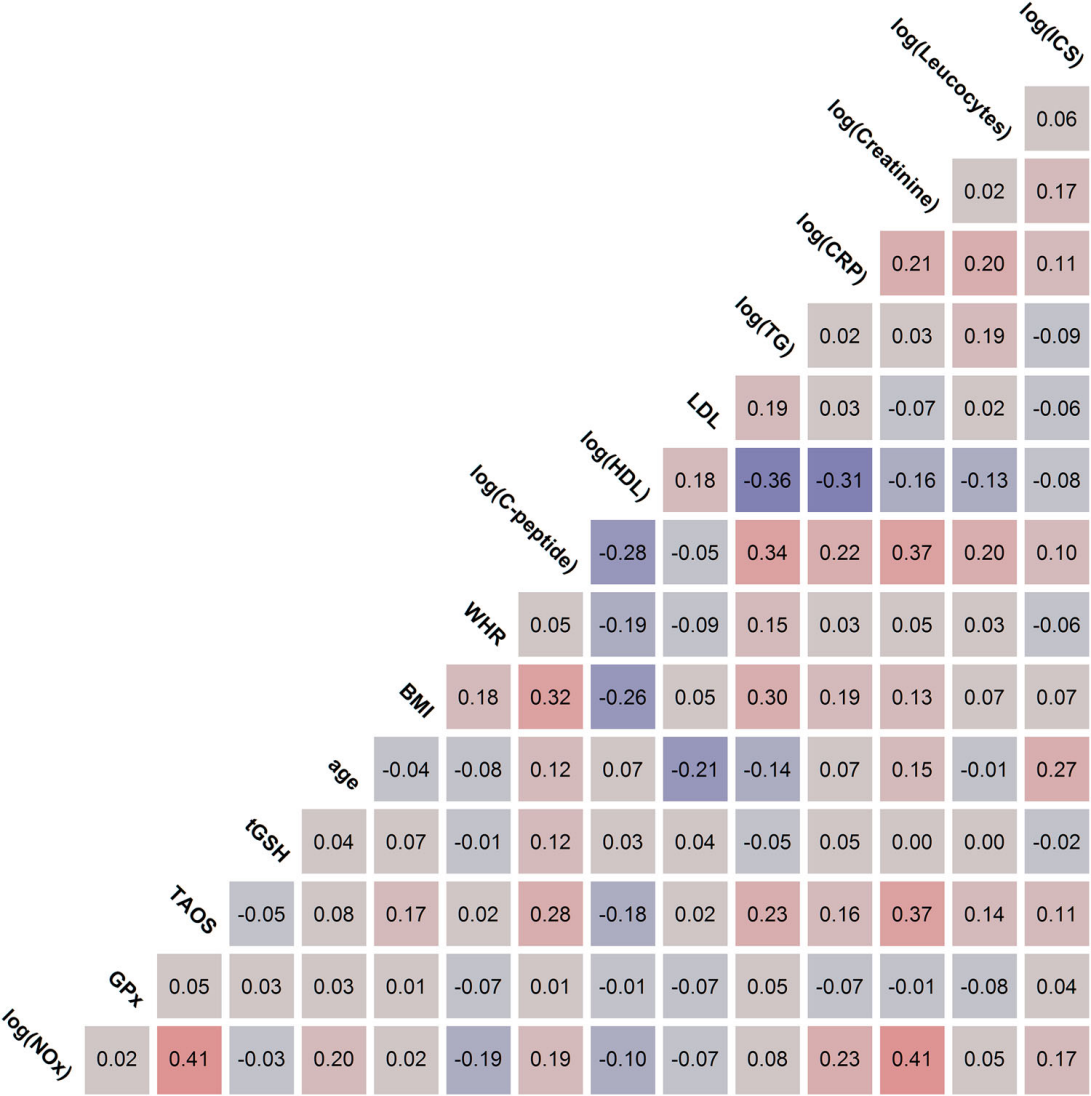
Pearson correlations of redox and metabolic parameters. Color saturation indicates the strength of the correlation. Abbreviations used: BMI: body mass index; CRP: C-reactive protein; GPx: glutathione peroxidases; HDL: high-density lipoprotein; ICS: intensive care stay; LDL: low-density lipoprotein; MI: myocardial infarction; NOx: nitrate/nitrite; PH: pulmonary hypertension; TAOS: total antioxidant status; TG: triglycerides; tGSH: total glutathione; WHR: waist–hip ratio.

In the next step, the Pearson correlations of the four redox parameters with clinical parameters of particular importance for patients with CVDs were analyzed. The strength of correlation was classified as ‘no correlation’ for |*r*|≤0.1, ‘weak’ for 0.1<|*r*|≤0.3, ‘moderate’ for 0.3<|*r*|≤0.5 and ‘strong’ for |*r*|>0.5. Figure 4 shows the Pearson correlations as determined for NOx levels, GPx activity, TAOS, total glutathione concentrations, age, BMI, WHR, C-peptide, HDL, LDL, TG, CRP, creatinine, leucocytes, and the time of ICS. Strong red colors indicate strong positive correlations; strong purple colors indicate strong negative correlations. Based on the data, total glutathione concentrations showed a weak correlation with C-peptide (+0.12). No correlation was found with any other clinical or redox parameters. TAOS moderately correlated with creatinine (+0.37). C-peptide (+0.28), TG (+0.23), HDL (−0.18), BMI (+0.17), CRP (+0.16), and leucocytes (+0.14), and ICS (+0.11) showed weak correlations. For GPx activity, no major correlation with other parameters in this study was found. NOx levels showed a moderate correlation with creatinine (+0.41) and TAOS (+0.41). A weak correlation was found between NOx and CRP (+0.23), age (+0.20), WHR (−0.19), C-peptide (+0.19), and ICS (+0.17). The figure enables searching for correlations between other parameters in addition to the redox ones.

**Figure 5.**
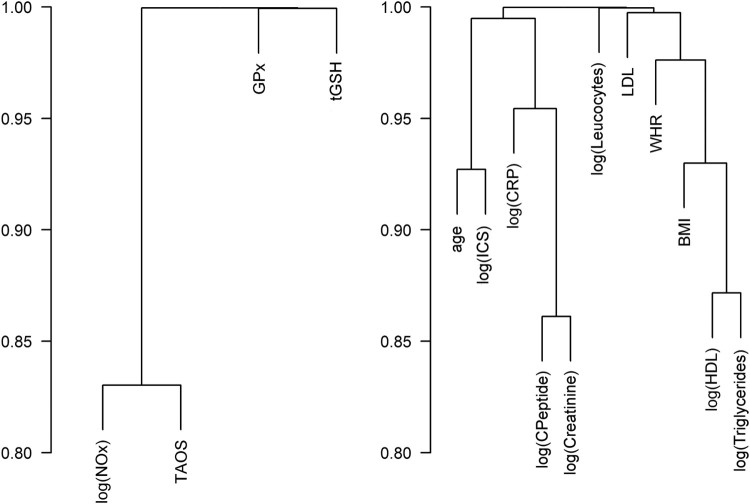
Cluster analyses of the correlations. Abbreviations used: BMI: body mass index; CRP: C-reactive protein; GPx: glutathione peroxidases; HDL: high-density lipoprotein; ICS: intensive care stay; LDL: low-density lipoprotein; NOx: nitrate/nitrite; TAOS: total antioxidant status; tGSH: total glutathione; WHR: waist–hip ratio.

Figure 5 shows a cluster analysis of correlations between the responses provided on the left side. No distinct pairwise correlation was found for the four redox parameters; however, a slightly positive correlation existed between TAOS and NOx. The cluster analysis of variables on the right side showed a stronger correlation between C-peptide and creatinine, between HDL and TG, and between age and duration of the ICS.

### Linear regression models for TAOS, total glutathione concentrations, GPx activity, and NOx levels

Table 2 gives an overview of all variables that contributed significantly to the explanation of the variation in the four linear regression models. The higher the percentages of explanation of variation the better the variables in the models explain the redox parameters.

**Table 2.** Overview of all model variables and their significance.

TAOS	tGSH	GPx	NOx
Log_2_(creatinine)*** ↑	Log_2_(C-peptide)** ↑	Smoking** ↓	Log_2_(creatinine)*** ↑
Log_2_(TG)*** ↑	COPD:CCB* ↓	Log_2_(TG)* ↑	Gender Female*** ↑
PH*** ↑	CCB* ↑	LDL^#^	ACE inhibitors*** ↓
COPD:CCB** ↑	Log_2_(TG)* ↓	PH^#^ ↑	ACEinh:Log_2_(HDL)*** ↑
Ventr. arrhyth.* ↑	Ventr. arrhyth.^#^ ↑		Ventr. arrhyth.*** ↑
			Diabetes** ↑
			Log_2_(CRP)** ↑
			Log_2_(HDL)** ↓
		PH* ↑	
		LDL^#^	
		COPD:CCB ^#^ ↑	
		COPD^#^ ↑	
		CCB^#^ ↓	
		Smoking^#^ ↓	
		Log_2_(TG)^#^ ↑	

Abbreviations used are. ACE: angiotensin-converting enzyme; CCB: calcium channel blocker; COPD: chronic obstructive pulmonary disease; CRP: C-reactive protein; GPx: glutathione peroxidases; HDL: high-density lipoprotein; LDL: low-density lipoprotein; NOx: reactive nitrogen species; PH: pulmonary hypertension; TAOS: total antioxidant status; TG: triglycerides; tGSH: total (reduced and oxidized) glutathione. Variables connected with a colon indicate statistical interaction of the variables.

The predictors are arranged according to their significance levels in the linear models. *** = highly significant (0 < *p* < 0.001), ** = very significant (0.001 < *p* < 0.01), and * = significant (0.01 < *p* < 0.05). *P*-values between 0.05 and 0.1 are marked with #. ↑ and ↓ indicate whether the predictor increases or decreases the redox parameter. Smoking, for instance, decreases GPx activity, while triglycerides increase it.

The linear regression model for TAOS accounted for 24.9% of the variation. According to our analyses, highly significant predictors for TAOS were creatinine (*p* = 1.04e-09), TG (*p* = 1.05e-06), and PH (*p* = 0.0006). The interaction between COPDs and CCBs was a very significant (*p* = 0.001) predictor, and ventricular arrhythmia was a significant predictor for TAOS.

The model explained 4.45% of the variation of total glutathione concentrations with the tested parameters. There was a very significant relation to C-peptide concentrations (*p* = 0.001) and a significant relation to TG levels (*p* = 0.029), CCB intake (*p* = 0.018), and the interaction of CCBs with COPD (*p* = 0.013).

The explained variance for GPx activity was 2.96%. A very significant predictor of GPx activity was smoking (*p* = 0.003), and the relation to TG (*p* = 0.028) was significant.

The model for NOx levels explained 33% of the variation. There was a highly significant relationship between NOx levels and gender (*p* = 3.13e-06), creatinine (*p* = 3.96e-15), ventricular arrhythmia (*p* = 9.49e-05), ACE inhibitors (*p* = 4.23e-05), and the interaction between ACE inhibitors and HDL (*p* = 4.30e-05). A very significant relation to diabetes (*p* = 0.001), CRP (*p* = 0.005), and HDL (*p* = 0.007) was also found. PH seemed to be a significant predictor of NOx levels (*p* = 0.033). Because NOx was logarithmized for the model, the estimates had to be delogarithmized in order to obtain the changes in NOx.

## Discussion

The objective of our study was to assess four major redox parameters (total glutathione concentrations, TAOS, GPx activity, and NOx levels) and their correlation with other clinically relevant parameters in blood from 500 patients directly before a cardiosurgical intervention. Patients were recruited from September 2011 to April 2013 at the Department of Cardiovascular Surgery, Justus Liebig University, in Giessen, Germany. All patients were admitted to the hospital for elective cardiac surgery; all patients were over 30 years old and had adequate German language skills to provide fully informed written consent. In order to efficiently and reliably determine the four redox parameters in quadruplicate in 500 samples, all redox assays were transferred to and optimized for the microplate reader (see methods section). To gain insight into the relationship between redox status and relevant parameters of CVDs, all parameters determined were statistically analyzed.

### Creatinine and PH are significant predictors of TAOS

In our patient group, plasma TAOS levels from 175 to 895 μM with a mean of 555 ± 107 μM were measured (Table 1). Taking into account that we studied patients with heart diseases, the values are in the range of previously reported data. Levels for healthy individuals reported in other studies range from 719 ± 0.260 µM to 1,670 ± 130 µM [31,13,32], although they were determined with various methods that correlate only partially [33]. Measuring the antioxidative capacity of blood plasma can be useful because several interacting antioxidant components such as total protein, uric acid, bilirubin, carotinoids, tocopherols, ascorbic acid, and unknown components are assessed simultaneously [12,13,34,35]. However, it should be kept in mind that the ABTS radicals, which are quenched in the assay, do not resemble physiological radical species [36].

In our patient cohort, the highest, positive correlation was found between TAOS and NOx (+0.41). As indicated by our model, this might partially be due to the fact that both parameters correlate with creatinine (+0.37 and + 0.41). Creatinine was also found to be a highly significant predictor of TAOS levels in the linear regression model. Elevated creatinine levels can result from kidney diseases, the intake of drugs that lower blood pressure, or potentially enhanced NOx levels that lead to vasodilatation. The resultant decreased glomerular filtration rate can lead to increased creatinine levels up to 3.1–5 mg per deciliter [37,38]. Creatinine levels in patients examined in this study varied from 0.4 to 6.0 mg per deciliter (mean ± SD: 1.04 ± 0.56), with reference levels for healthy persons being 0.6 to 1.2 mg/dl for women and 0.7 to 1.3 mg/dl for men (internal reference values of UKGM, Giessen). Our data are in accordance with the results of Lamont et al. [34], who found a correlation between TAOS and creatinine (0.256) and an even higher correlation with uric acid (0.526).

In our study, the intake of several drugs that lower blood pressure, namely ARBs, ACE inhibitors, CCBs, and beta-blockers, was assessed in the form of categorical parameters. Interestingly, CCBs in combination with the presence of COPD showed a significant relation to high TAOS (Table 2). Some previous studies found that CCBs may have antioxidant effects [39], whereas others did not support these findings [40]. Interestingly, both CCBs and ACE inhibitors were negative predictors for NOx, which might be explained by the fact that lowered blood pressure due to medication does not require high NOx levels.

Although PH was very rare in this study and therefore its influence cannot be interpreted reliably, it seemed to have a highly significant influence on TAOS. One possible explanation for this phenomenon is the mobilization of antioxidants such as vitamin E [41]. After lung injury the activation of signal transduction pathways via protein oxidation and lipid peroxidation might lead to enhanced vitamin E recruitment from body stores. Type II lung cells, which are able to regulate the turnover of vitamin E, are suggested to contribute to this [41]. However, a putative influence has to be confirmed in studies with higher numbers of patients suffering from PH. In addition to PH, ventricular arrhythmia (low significance) and TG levels (high significance) were identified as positive predictors for TAOS.

It should be emphasized that the statistical model presented here for TAOS explains 24.9% of the variance. Therefore, around 75% of it remains unexplained. Possible further predictors with significant impact that have not been assessed in our study include not only protein levels, uric acid, bilirubin, and antioxidant vitamins [12,13], but also currently unknown plasma components and pathophysiological conditions.

### Total glutathione levels are linked to C-peptide and CCBs

In our patients, total glutathione levels in whole blood ranged from 0.11 to 1.11 mM (mean ± SD: 0.61 ± 0.19 mM), and erythrocyte levels from 0.34 to 2.83 mM (mean ± SD: 1.52 ± 0.48 mM) were determined. In a previous study that employed the same method, a mean total glutathione level of 0.78 ± 0.21 mM in the whole blood of healthy African children was determined [42]. Pastore et al. [43] reported mean total glutathione levels in blood from 0.34 up to 1.22 mM determined in different studies and with different methods. Michelet et al. [44] reported that in other studies, total glutathione concentrations from 684 μM in whole blood samples to 2,525 μM in red blood cells were measured, and correlations with age and/or gender were often found. In their own study, however, they did not find differences between men and women, which our results confirm.

In our study, glutathione was found to correlate only with C-peptide (+0.12). The antioxidant system in erythrocytes from diabetic persons was previously shown to be impaired, and reduced GSH concentrations were also found [45]. C-peptide levels are also lowered in diabetic patients, which may explain the correlation. As summarized in Dröge [46], increased glucose levels are associated with elevated ROS production; therefore, elevated ROS levels are often found in diabetic patients. The linear regression model presented here confirmed the relationship between C-peptide levels and total glutathione concentrations (Figure 4) and found C-peptide to be a very significant predictor of total glutathione (Table 2). Diabetes was not identified as a predictor of glutathione levels; however, TG levels were strong predictors (Table 2). This might be caused by constellations related to the metabolic syndrome, which is associated with hypertension, obesity, elevated glucose levels, low HDL levels, and high TG and is often found in diabetic patients. Two other significant predictors for glutathione were the intake of CCBs (positive predictor) and their intake in combination with COPD (negative predictor). These correlations are discussed below in an extra paragraph dedicated to drug effects on redox parameters.

Concerning the linear regression model, it is important to mention that the parameters assessed only explain around 4.45% of the variance in tGSH levels. Other parameters such as nutritional status, infections, inflammation, malignancies, circadian rhythms, or polymorphisms of γ-glutamyl-cysteine synthetase, which were not the focus of our study, are likely to further contribute to the variation [43].

### Glutathione peroxidase activity is influenced by smoking

In our patients, mean activity of the selenoprotein GPx (assayed with cumene hydroperoxide as a substrate) was determined to be 110 ± 3 U/g Hb, ranging from 4 to 220 U/g Hb. Levels in whole blood ranged from 5.69 to 32.3 U/ml, with a mean of 15.6 ± 4.26 U/ml. These data are in the same range as previously reported activities, although direct comparison is again difficult due to varying assay setups that include various peroxides employed as substrates. A mean GPx activity of 22.2 ± 5.9 U/g Hb was found in the erythrocytes of healthy persons by using *tert*-butyl hydroperoxide as a substrate [47]. Habdous et al. [31] measured mean levels of 51.5 ± 13.0 U/g Hb and 55.0 ± 16.1 U/g Hb in women, and another study with the elderly found mean levels of 497 ± 166 U/l [1]. In yet another study, mean GPx-1 activity of 49.2 ± 11.6 U/g Hb in erythrocytes was found, ranging from 7.4–99.6 U/g Hb, whereas mean levels in patients with cardiovascular events (45.3 ± 12.9 U/g Hb) were lower than those without (49.8 ± 11.3 U/g Hb) [16].

In our study, smoking significantly decreased GPx activity according to the linear regression model. This was also reported by Blankenberg et al. [19], who found associations of GPx activity with CVDs when they evaluated whether enhanced activity of GPx-1 can protect against cardiovascular events. Individuals with non-fatal MI and those who had died from cardiac diseases had significantly lower GPx-1 baseline levels. Death via non-cardiovascular causes did not lead to different baseline levels of GPx activity compared to event-free patients. When evaluating the effect of medication on CVDs, only statins had a minor, non-significant influence. Patients taking statins more often appeared in the highest quartile of GPx-1 activity [19]. The second significant predictor of GPx activity in our model was the TG level. The model predicts an increase of 0.05 U/mg Hb if TG levels double. Although this increase is small, a possible explanation could be that GPx-4, a phospholipid hydroperoxidase that protects lipids from oxidation, is upregulated with high TG levels.

Moreover it is important to note that only 2.96% of the variance in GPx activity levels is explained by the model. This means that about 97% is the result of parameters that were not in the model such as selenium intake, physical exercise, and genetic poly­mor­phisms. Conflicting findings on the relationship between GPx activity and factors such as age and gender can be found in literature and were summarized by Habdous et al. [31].

Notably, dietary selenium intake closely regulates GPx-1 activity in the normal range by influencing selenocysteine availability. This is due to the fact that GPx mRNA is unstable at low selenocysteine levels [48] and that GPx are not or are only rarely catalytically active without incorporated selenocysteine. Arthur warned against relating small changes in blood GPx activity to the oxidative mechanisms for disease pathogenesis [49]. In severe selenium deficiency, GPx activity decreased to less than 1% of the control, and yet no evidence of oxidative damage or pathology was found. This underscores the strong dependence of GPx on and regulation by selenium availability. Changes in activity are not always related to oxidative damage, which is most likely due to compensation by other peroxide-detoxifying proteins such as peroxiredoxins and antioxidants. Nevertheless, several studies could show that changes in selenium status affect, for instance, isoprostane F2α concentrations, the production of eicosanoid metabolites, and compounds associated with heart diseases [50]. Associations between GPx-1 activity and cardiac events were found in mice overexpressing GPx-1; cell damage caused by ischemia in the heart was decreased; and recovery of contractile force was better upon GPx-1 overexpression. Decreased infarct sizes and a significantly decreased release of creatine kinase were found. The opposite was reported for GPx-1-deficient mice. GPx-1 appears to have an important role in protecting against ischemia reperfusion injuries [51].

### Nitrate and nitrite levels are influenced by creatinine, gender, ACE inhibitors, and ventricular arrhythmia

NOx (nitrate and nitrite) levels observed in our study ranged from 8.79 to 183 μM, with a mean of 35.9 ± 25.0 μM. These data are comparable to results from Sastry, who used ${\text{ZnSO}}_{4}^{-}$ for protein precipitation, as we did in our study, and found average levels of 43.5 ± 4.42 μM for nitrate and 3.68 ± 0.56 μM for nitrite [29]. Comparing these results to absolute levels found in other studies is challenging, for there are many different methods and influencing factors. In their review Tsikas et al. give an excellent summary of previous studies using the Griess reaction [18]. Major differences in plasma/serum levels were found between the studies, e.g. from 0 to 42 µM and from 13 to 108 µM, respectively.

In our approach, strong correlations of nitrate/nitrite levels were found with creatinine levels (+0.41) and TAOS (+0.41). As described above, the correlation with TAOS is assumed to be confounded by creatinine. NO directly regulates renal function by modulating vascular tone and can be eliminated via glomerular filtration. High creatinine levels indicate renal dysfunction or limited perfusion. A study of patients with chronic renal diseases also found a significant positive correlation between serum NO and creatinine levels (*r* = 0.81) [52]. Furthermore, in our study, CRP showed a positive correlation with NOx levels (+0.23). Another study found CRP to be a predictor of NO levels in patients with CAD, although they detected an impairment of NO bioavailability [53]. In contrast, systemic inflammatory disorders (e.g. sepsis) greatly increase iNOS activity [20]. In our study, weak correlations were further found with age (+0.20), WHR (−0.19), C-peptide levels (+0.19), and ICS (+0.17). In a study with murine macrophage cells, C-peptide stimulated nitrite generation [54].

The linear regression model for NOx explains 33% of the variance. According to the model, many parameters have significant (PH), very significant (HDL, CRP, diabetes), and highly significant (ventricular arrhythmia, interaction between ACE inhibitors and HDL, ACE inhibitors, being female, creatinine) influence on NOx levels. Hyperglycemia inhibits eNOS; therefore, diabetes may be a significant predictor influencing NO levels [55]. It should be noted in this context that further factors with an influence on NOx levels, which were not systematically assessed here, include e.g. exercise, diet, circadian rhythm, and inflammation [56]. Notably, particular effort has recently been devoted to understanding how redox signaling may contribute to vascular pathobiology in human hypertension. As summarized in Togliatto et al. [57], the decrease of NO levels, the antioxidant activity often found in preclinical models of hypertension, and the ability of antioxidant approaches to reduce ROS levels have stimulated clinical studies on the contribution of ROS in humans.

### The influence of medication on redox status

Interestingly, a number of significant interactions were observed between the medication of the patients and the measured redox parameters. In our study, the intake of several drugs that lower blood pressure, namely ARBs, ACE inhibitors, CCBs, and beta-blockers, was assessed in the form of categorical parameters.

The model for total glutathione concentrations predicts increased total glutathione levels for persons taking CCBs, whereas the combination of COPD and CCBs decreases blood glutathione levels. Mak et al., 1992, found in a cell culture study that the loss of glutathione in cells after free radical stress in the form of superoxide and hydroxyl radicals can be inhibited by CCBs (65%–87%) [39]. CCBs are quite diverse, but all of them contain aromatic unsaturated ring structures, which are common in classic aromatic ‘chain breaking’ antioxidants. Furthermore, CCBs are lipophilic. It is assumed that these properties protect the membranes from lipid peroxidation. Although the mechanisms are not fully understood, the intake of CCBs may indeed lead to higher total glutathione levels [39]. According to our model, COPD together with CCBs decrease tGSH levels. Although COPD had been found to be linked to GSH deficiency in other studies [43], COPD alone did not reach levels of significance as a predictor of glutathione concentrations in our patients.

Furthermore, according to our model, ACE inhibitors very significantly decrease the NOx levels in the patients (Table 2). ACE inhibitors affect NOx levels by inhibiting the conversion of angiotensin I into the vasoconstrictive angiotensin II with simultaneous inhibition of the degradation of vasodilating bradykinine [55]. This vasodilation is likely to induce reduction of vasodilating NOx levels. Finally, CCBs were found to be negative predictors for NOx and COPD, and the combination of COPD and CCBs were positive predictors.

In this context, it is also of interest to mention that among all parameters tested, age showed the highest correlation to the duration of the ICS (0.27), followed by NOx levels (0.17), creatinine (0.17), TAOS (0.11), and CRP (0.11).

## Conclusions and outlook

Redox regulation and oxidative stress are essentially involved in the pathophysiology of CVDs, the metabolic state of patients before surgery, the surgical intervention itself, and the ensuing recovery phase. Here we identified correlations between markers of oxidative stress and other relevant physiological or pharmacological parameters such as the intake of CCBs and ACE inhibitors in our patients. We believe that it will be of great interest to follow up these results in focused clinical studies. The correlations determined here have the potential to contribute to a more differentiated diagnosis of cardiovascular disorders and their underlying or accompanying metabolic changes. Furthermore, single parameters or certain constellations of parameters might be useful for better predicting the outcome of a cardiosurgical intervention and/or the duration of the ICS. In the long term this knowledge might be valuable for the improvement of patient management. Finally, the alterations of glutathione homeostasis by CCBs and the decrease of NOx levels under ACE inhibitors should be followed up thoroughly. Since both drugs are usually applied as a long-term medication, they might lead to long-term changes in redox homeostasis. It is therefore important to understand whether the changes represent adaptive mechanisms or potential side effects.
